# Notes of Hospital Clinics

**Published:** 1897-07-17

**Authors:** 


### Notes of Hospital Clinics.

[*Reported for* The Hospital, *and corrected by the authors.*]

### ST. THOMAS'S HOSPITAL.

### Clinical Lecture on Two Cases of Muscular Atrophy.

By J. F. Payne, M.D., F.R.C.P., Physician to the Hospital.

Two cases of muscular atrophy were described, in one of which the atrophy was primary, and in the other was of nervous origin.

The first case, which was shown, was that of a boy aged 14, who had been unable to walk for many years, and had had obstinate constipation during the last 12 months. There was extreme wasting of the muscles, which was especially well marked in those of the trunk, shoulder, upper arm, and thigh; the muscles of the neck, forearm, hand, calf, and foot being much less affected. The muscles of the face, the larynx, and the diaphragm were entirely unaffected. There was no anæsthesia, pain, or spasm. There were puffy swellings in the serratus, sterno-mastoid, and infra-spinatus, besides relative enlargement of the muscles of the calf. The patient breathed wholly with the diaphragm, owing to wasting of the muscles attached to chest wall, and his intestines were loaded with fæcal masses, owing to wasting of the muscles of the abdominal wall. The case was described as an example of primary muscular atrophy, unconnected with disease of the nervous system, which probably began in the muscles of the trunk and spread outwards. Of this there were two varieties. Variety 1: The connective and adipose tissue is increased (the so-called pseudo-hypertrophic paralysis). Variety 2: There is no increase of these tissues. Different as these two types might appear, the lecturer was of opinion that there was no strict dividing line between them, and he considered that this case had probably been one of the first variety, the pseudo-hypertrophic form, at the beginning (as shown by the puffy swellings still remaining in one or two muscles, and the roundness of the calf), and had gradually become assimilated to the second variety. He thought that this often occurred. The cause of the disease was quite unknown.

The second case was that of a man, aged 40, who two years ago fell from a horse, the horse then falling on him. Three months later he had twitchings in the right hand, and then three months after this he had twitchings in the left hand. Then, later on, he had twitchings in the feet, and pain and cramp in the legs. He was a healthy-looking man, with muscular wasting, affecting especially the hands on both aspects, and also the deltoid and scapular muscles. The legs were less affected, and the trunk muscles at present not at all. The knee-jerks were exaggerated, and ankle clonus was present. Speech was normal. There was no anæsthesia. Fibrillar tremors occurred in the wasted muscles. The legs were in a condition of spastic paraplegia. This was a case of so-called progressive muscular atrophy or wasting palsy, a condition in which there are the following constant changes in the nervous system: (*a*) Degeneration of cells of the anterior cornua, causing atrophy of muscles; (*b*) degeneration of pyramidal tracts, causing spastic symptoms. The causes were shock or fright, cold, and injury concurrently with syphilis.

The points of interest about these two cases were that Case 1 was an example of a developmental disease, while Case 2 was an example of a disease due to injury. The differential diagnosis might be made by noticing the order in which the muscles become affected, and in most cases by the presence or absence of fibrillar tremors in the affected muscles. Examples like the first case were rare, while examples similar to Case 2 were comparatively common. As to treatment: Case 1 would require strychnine, and massage if the wasting were not too great; while Case 2 would be benefited by the constant current if the wasting had not gone too far.

### HOSPITAL FOR CONSUMPTION AND DISEASES OF THE CHEST, BROMPTON.

### Clinical Lecture on Aortic Stenosis.

By Sidney Martin, M.D., F.R.S., Assistant Physician to the Hospital.

Seven cases were shown illustrating the conditions which are apt to be met with, and the complications found in conjunction with stenosis of the aortic and pulmonary orifices.

Case 1 was a girl aged 19, whose left ventricle was slightly dilated and whose right ventricle was greatly hypertrophied, and over whose second, third, and fourth right costal interspaces, near the margin of the sternum, a systolic thrill was felt and a rough systolic bruit heard. This was a case of aortic stenosis, the hypertrophy of the right ventricle being probably due to associated mitral regurgitation.

Case 2 was a man aged 38, who had a rough systolic bruit over the aortic cartilage, and a short musical systolic murmur at the apex. There was great hypertrophy of the left ventricle and enlargement of the right heart. The condition was aortic stenosis and mitral constriction.

Case 3 was a boy, aged eight, in whom a systolic and diastolic murmur were heard at the base and a systolic murmur at the apex. These three cases illustrated the frequent association of aortic and mitral disease.

Case 4 was a boy, aged six, who had hypertrophy of both right and left ventricles, and over whose first three left intercostal spaces a double thrill was felt and a double murmur heard. This case illustrated congenital disease of pulmonary valves, the hypertrophy of the left ventricle being probably due to there being a communication between the two ventricles.

Case 5 was a man, aged 32, with greatly hypertrophied right ventricle and a systolic bruit over the pulmonary cartilage. The left ventricle was not enlarged. There was recent tuberculosis in the upper lobe of the right lung. This case illustrated pulmonary stenosis associated with phthisis; phthisis being a common termination of these cases.

Case 6 was a man aged 40 who had a systolic bruit at the base, loudest on the right of the sternum, together with hypertrophy of the left ventricle, the second sound of the heart being absent. This was a case of uncomplicated aortic stenosis.

Case 7 was a man aged 72, who had a rough musical systolic bruit most marked at the aortic orifice, but conducted all over the cardiac area, the second sound being absent. He had never had rheumatism. This case was an example of slowly progressing atheroma of the valves, leading to cohesion of the cusps. The musical bruit showed that the valves were stiffened.

In commenting on these cases Dr. Martin said that many people had slight aortic stenosis for years without being aware of it, or of anything being the matter with them. It had been shown experimentally that with slight degrees of stenosis the left ventricle worked harder, and so kept up the circulation fairly well until some extra stress was thrown upon it.

In uncomplicated cases of aortic stenosis the left ventricle alone was hypertrophied; but if mitral stenosis was also present there might be hypertrophy of the right ventricle without any hypertrophy of the left, because so little blood was entering it. But if mitral regurgitation also were present, then the left ventricle became hypertrophied. With pure pulmonary stenosis there was, of course, hypertrophy of the right ventricle only. Patients with aortic stenosis were usually pale and wasted, and complained of dyspnœa, but not of œdema. Death in these cases occurred either by angina pectoris, by failure of the left ventricle, or by degeneration of the muscular substance. It should be remembered that the changes which led to narrowing of the aortic valves were in many cases only a part of a much more widely-spread atheromatous affection, which often affected the nutrient arteries of the cardiac muscle. The degeneration of the muscular walls which occurred in connection with aortic stenosis was therefore often produced in a somewhat different manner from that which occurred in connection with diseases of other valves.

